# Testing syndemic models along pathways to psychotic spectrum disorder: implications for population-level preventive interventions

**DOI:** 10.1017/S0033291725000455

**Published:** 2025-03-13

**Authors:** Yamin Zhang, Jeremy Coid

**Affiliations:** 1 Department of Prevention and Control, Affiliated Mental Health Center & Hangzhou Seventh People’s Hospital, Zhejiang University School of Medicine, Hangzhou, China; 2 Liangzhu Laboratory, MOE Frontier Science Center for Brain Science and Brain-machine Integration, State Key Laboratory of Brain-machine Intelligence, Zhejiang University, Hangzhou, China; 3 Wolfson Institute of Population Health, Queen Mary University of London, London, UK

**Keywords:** childhood adversity, psychotic spectrum disorder, risky sexual behavior, substance misuse, syndemic, violence/criminality

## Abstract

**Background:**

Population-level preventive interventions are urgently needed and may be effective for psychosis due to social determinants. We tested three syndemic models along pathways from childhood adversity (CA) to psychotic spectrum disorder (PSD) and their implications for prevention.

**Methods:**

Cross-sectional data from 7461 British men surveyed in 5 population subgroups. We tested interactions on both additive and multiplicative scales for a syndemic of violence/criminality (VC), sexual behavior (SH), and substance misuse (SM) according to the presence of CA and adult traumatic life events; mediation analysis of path models; and partial least squares path modeling, with PSD as outcome.

**Results:**

Multiplicative synergistic interactions were found between VC, SH, and SM among men, who experienced CA and traumatic adult life events. However, when disaggregated, only SM mediated the pathway from CA to PSD. Path modeling showed traumatic life events acted on PSD through the syndemic and had no direct effect on PSD. Higher syndemic scores and living in areas of deprivation characterized men with PSD and CA.

**Conclusions:**

Our findings support a broad division of PSD into cases due to (i) biological/inherent causes, and (ii) social determinants, the latter including a syndemic pathway determined by CA. Preventive strategies should focus primarily on preventing adverse effects of CA on developmental pathways which result in PSD. Single component prevention strategies may prevent triggering effects of SM on PSD during adolescence/early adulthood among vulnerable individuals due to CA. Future research should determine applicability and transferability of interventions based on these findings to different populations, specifically those experiencing syndemics.

## Introduction

Recent developments in syndemic theory have suggested new approaches to public health preventive interventions (Chakrapani et al., 2019; Tsai, 2018; Tsai, Mendenhall, Trostle, & Kawachi, 2017). These are urgently needed in psychiatry, particularly for psychotic illness (Zahid, Hosang, de Freitas, Mooney, & Bhui, 2023). Syndemic research has suggested a plausible environmental rather than biological explanation for the increased risk of psychosis observed among black and other disadvantaged men living in Hackney, an inner-urban London borough, through a mechanism of multiplicative interactions between substance misuse (SM), violence/criminality (VC), and poor sexual health (SH) (Coid et al., 2020). The implication is that population-level interventions would need to be targeted in similar urban areas and among sub-groups experiencing a syndemic rather than applied universally. Subsequent study indicated that synergy between these three syndemic components ultimately depends on childhood adversity (CA) to have a specific impact on psychotic spectrum disorder (PSD) and to differentiate PSD from other psychopathological outcomes, such as depression or anxiety disorder (Zhang & Coid, 2024). Direct and indirect pathways were found from CA to PSD, with an indirect pathway involving the syndemic having greatest impact. However, because not all PSD cases experience CA, this means there must be additional pathways to psychosis determined primarily by biological or inherent causes.

Psychopathological changes in brain development secondary to social determinants are likely to differ along these pathways, with multiple adverse social and environmental exposures causing stress from infancy, which impacts on brain development (McLaughlin, Weissman, & Bitran, 2019; Pruessner, Cullen, Aas, & Walker, 2017; Teicher, Samson, Anderson, & Ohashi, 2016). Confirmation of this division into two major pathways or broad subtypes (biological/inherent causes – social determinants) would imply differing preventive and treatment strategies will be necessary, delivered at different developmental stages, possibly targeted at different population sub-groups, residing in different geographical areas.

In this study, we aimed to test Tsai’s syndemic models (Tsai et al., 2017, Tsai, 2018), develop preliminary hypotheses, and demonstrate their implications for new population level interventions for the subgroup of PSD due to social determinants.

## Testing Tsai’s syndemic models

Syndemic theory can be applied to individuals but is principally about population health and explains clustering of various forms of psychopathology, physical health conditions, and health-related behaviors in populations through harmful social factors, which mutually exacerbate each other and synergistically amplify the disease burden (Singer, 2009; Tsai et al., 2017). It is important in syndemic research to show firstly disease concentration, where two or more epidemics co-occur in particular temporal or geographical contexts due to harmful social conditions. Secondly, synergistic interactions between these epidemics which mutually reinforce each other to amplify the disease burden. Thirdly, the social and economic contexts in which these occur because they can foster and exacerbate clusters of disease and adverse health-related behaviors and may differ considerably between different contexts (Mendenhall, Kohrt, Norris, Ndetei, & Prabhakaran, 2017). Population interventions will then involve either eliminating one or more components of the syndemic or by lessening their impact.

Tsai (2018) argued that syndemic interaction can be applied to three different models of co-occurring epidemics with implications for preventive population interventions: mutually causal epidemics, synergistically interacting epidemics, and serially causal epidemics. Using these models, research should firstly rule out hypothesized causes with no direct effects on outcome. However, these should still be investigated because they may have indirect effects which need careful consideration in any proposed intervention. Secondly, they may help determine whether a single or multi-component intervention is chosen, with implications for logistics and cost. For example, if the syndemic is characterized by synergistically interacting epidemics that are not mutually causal, then implementing a single-component intervention may still be effective (Tsai et al., 2017). Thirdly, for serially causal epidemics, different interventions would be needed based on models of life course epidemiology (Tsai, 2018).

Chakrapani et al. (2019) were first to simultaneously test these three models in a study of men who have sex with men (MSM). The dominant model of syndemic interactions was confirmed, with partial support for models of serially causal and mutually causal epidemics. There are currently only three syndemic studies with psychosis as outcome and the three models have never been simultaneously tested: Coid et al. (2020) demonstrated synergistic interactions but did not investigate Tsai’s other two models. Bhui, Halvorsrud, Mooney and Hosang (2021) confirmed a model of mutually causal epidemics but did not demonstrate synergistic interactions. Zhang and Coid (2024) confirmed synergistic interactions and a model of mutually causal epidemics but did not investigate a serially causal model.

The overarching aims of the paper were to test three models (Tsai, 2018) using a previously developed latent class, categorical outcome of PSD (Zhang & Coid, 2024), then identify their implications for future population-level interventions. Because our data are cross-sectional, the study cannot demonstrate causality as implied by Tsai (2018). Our model testing must therefore be considered prototypical, requiring future longitudinal study. Nevertheless, we used the same analytical method of Chakrapani et al. (2019) (whose data were also cross-sectional) with specific aims of testing: (i) a model of synergistically interacting epidemics by evaluating joint associations between SM, VC, and SH on our psychosis outcome and assessing for synergistic interactions on both additive and multiplicative scales; (ii) the model of serially causal epidemics (or ‘chains of risk’) (Coie et al., 1993) in which a syndemic score and number of adult traumatic life events were each conceptualized as potential mediators of effects of CA on the PSD outcome; (iii) a model of mutually causal epidemics (Singer, 1996) in which two of the three exposures hypothesized as contributing to psychosis were conceptualized as mutually causal, with the syndemic leading to adult traumatic events and adult traumatic events leading to the syndemic; and (iv) we have previously proposed that etiological mechanisms follow different pathways to psychosis which can be broadly split into (a) those with biological or inherent causes and (b) those involving social determinants with synergistic interactions between specific social factors (Zhang & Coid, 2024). We finally compared the prevalence of these two primary etiological pathways according to contextual variables and across different population subgroups to guide the future targeting of population-level interventions.

## Methods

### Study participants

We used data from the Second Men’s Modern Lifestyles Survey carried out in 2011 using random location sampling (RLS), an advanced form of quota sampling shown to reduce biases when interviewers are able to choose locations to sample from. These surveys were originally intended to study violence among men. Sub-populations were chosen for comparison with a general population sample, including those from specific geographical areas based on high levels of socio-economic deprivation, ethnic mix, and likelihood these would have a high prevalence of violence. Each population cross-sectional survey sample was based on the national census for British men. RLS was used because of its effectiveness with hard-to-reach populations and where there is often high attrition in conventional surveys for men with demographic characteristics and key psychopathology of interest in studying violence (see supplementary file).

Surveys included representative samples of all men in England, Scotland, and Wales; a boost sample of Black and minority (BME) men; a boost survey of unemployed and lower social status; and two additional surveys conducted in Glasgow East, Scotland, and Hackney, East London, areas of exceptionally high socioeconomic deprivation, substance misuse, crime, violence, and social exclusion. Individual sampling units (census areas of 150 households) were randomly selected for each survey within British regions (England, Scotland, and Wales) in proportion to the population. Identical sampling principles and instruments were used for each survey (see supplementary file).

A self-administered questionnaire piloted in a previous survey was adapted in which the men self-reported their lived experiences over the lifespan, together with standardized instruments to measure psychopathology. Informed consent was obtained from survey respondents who completed the pencil and paper questionnaire in private and were paid £5 for participation.

The study was approved by the Queen Mary University of London Ethics Committee. The authors assert that all procedures contributing to this work comply with the ethical standards of the relevant national and institutional committees on human experimentation and with the Helsinki Declaration of 1975, as revised in 2008.

## Measures

### Environmental exposures

We measured self-reported adverse childhood experiences (ACEs) before age 16 years: bullying, witnessing violence in the home, sexual abuse/assault, physical abuse, neglect, serious illness/injury, and being placed in a children’s home or foster care. We scored total factors from 0 to 7.

We measured adult traumatic life events after age 16 years): experiencing domestic violence, sexual assault, life threatening injury/illness, separation/divorce, fired from job, homelessness, and serious money problems. We scored total factors from 0 to 7.

### Syndemic score

Our choice of health-related measures was based on Singer’s (2009) theoretical approach, as described in the supplementary file. To obtain an overall syndemic score, we evaluated 15 health-related measures from three domains: s*exual health/risks*, defined as ≥10 sexual partners in the past year, contraceptive use rare/never, sex with prostitutes, anal sex, sex with men, forced sex on partners, and sexually transmitted infection; *substance misuse*, defined as alcohol or drug dependence, cannabis use more than four times weekly; *violence and criminality*, defined as friends encouraging criminal activity, weapon carrying, fear of violent victimization, intimate partner violence, and repeated assaults/fights.

The structure of these domains was validated and factor scores for SM, SH, and VC were obtained using confirmatory factor analysis, as previously described (Zhang & Coid, 2024). To investigate potential syndemic effects, the model was extended to allow for a higher-order latent variable originally representing a general syndemic dimension in the previous study and was used again in this study as a general syndemic score in our analyses, with separate or combined scores on the SM, SH, and VC domains.

### Psychiatric morbidity

Our outcome measure of PSD was partly derived from the Psychosis Screening Questionnaire (Bebbington & Nayani, 1995), which covers hypomania, thought interference, paranoid ideation, strange experiences, and auditory and visual hallucinations in the past year and which was included together with measures of anxiety and depression to derive four latent classes, as described in a previous study (Zhang & Coid, 2024). We used the same categorical construct of psychosis/anxiety for our measure of PSD.

The Hospital Anxiety and Depression Scale consists of seven items assessing anxiety and seven items assessing depression in the past week. Each of the seven items is scored from 0 to 3 with a higher score indicating a worse outcome (Zigmond & Snaith, 1983). We defined anxiety and depression based on scores ≥11. Scores ≥20 on the Alcohol Use Disorders Identification Test (Babor, Higgins-Biddle, Saunders, Monteiro, & Organization, 2001) and scores ≥25 on the Drug Use Identification Test (Berman, Bergman, Palmstierna, & Schlyter, 2005) were used to identify drug dependence, respectively. The Structured Clinical Interview for DSM-IV Personality Disorders Screening Questionnaire identified antisocial personality disorder (ASPD) (Coid & Ullrich, 2011).

The Standard Occupational Classification for the UK was used to measure social class (Surveys, 1991). Socioeconomic deprivation was measured using both a ranking and score based on the Index of Multiple Deprivation Ranking (IMDR) (McLennan et al., 2011).

### Statistical analysis

We followed the analytical method of Chakrapani et al. (2019) to test Tsai’s three models. To test the model of synergistically interacting epidemics, we tested interactions on both the additive and multiplicative scales (Rothman, 1974; Rothman, Greenland, & Walker, 1980) and according to presence or absence of both child adversity (ACEs > = 1) and adult traumatic life events (≥1). Although our primary hypothesis postulates that it is the multiplicative effect that is the key etiological factor in determining psychosis as outcome, it is also important to test for additive interactions when developing clinical prevention strategies as these highlight cumulative impact of risk factors.

To test the model of chains of risk (serially causal epidemics) we conducted a mediation analysis of path models utilizing R package ‘SEMinR’. Our approach utilized partial least squares path modeling (PLS-PM) due to its capability to accommodate both continuous and categorical variables. In our model, we focused on PSD as the binary outcome and the number of ACEs as the independent variable. Initially, we explored the syndemic score and number of adult traumatic events as potential mediators. Following the identification of significant mediation by total syndemic score, we investigated the mediating effect of the three component scores. To assess the significance of these mediations, we employed bootstrapping to estimate standard errors and calculate confidence intervals.

To test the model of mutually causal epidemics, PLS-PM with reciprocal arrows or feedback loops was used. The cyclic or non-recursive model tested is shown in Fig. 2. Given the temporal precedence of causes over effects, no arrow was directed from the exposures happening in adulthood to ACEs. Bidirectional arrows were specified between the syndemic score and number of adult traumatic events, both assessed within the same reference periods. The fit values of the path model were also estimated, with Alpha, rhoC, and rhoA >0.7, while AVE > 0.5 interpreted as indices of good fit.

## Results

There were a total of 7461 men from five surveys. Table S1 shows marked differences in demographic characteristics, psychosis, mean scores of ACEs, adult trauma, and general syndemic scores between the survey samples indicating necessary adjustments for age, ethnicity, single marital status, non-UK birth, IMDR, and survey type.

Table S2 shows that a categorical latent class of psychosis/anxiety co-occurred with suicide attempts, ASPD, cannabis misuse, alcohol, and drug dependence but not depressive disorder. All high-risk sexual behaviors, violence and criminality, and adverse child experiences were more prevalent among men with PSD. All adult traumatic events were more prevalent, except being fired from a job.

## Synergistically interacting epidemics

Table 1 shows that SM, VC, and SH were associated with PSD in a multivariate logistic regression model without product terms. We then tested for interactions between these three syndemic components and PSD on the multiplicative and additive scales. Additive interactions are clinically relevant when the focus is on population health and can help guide clinical prevention strategies. Multiplicative interactions focus on high-risk individuals with multiple risk factors to identify those in need of more rigorous clinical intervention but are also potentially indicative of underlying etiology.

**Table 1.** Association and synergy between syndemic components, child adversity, and adult traumatic events for latent class of PSD (n = 1623)

	Direct associations	Synergy between factors	
	OR (95% CI)	OR (95% CI)	RERI (95% CI)
**PSD (latent class)**			
SM	4.27 (3.61–5.04)^***^		
VC	3.47 (2.98–4.03)^***^		
SH	4.13 (3.50–4.87)^***^		
**Interactions (total sample)**			
SM by SH		1.19 (1.05–1.36)^**^	5.50 (−0.48–11.49)
SH by VC		1.18 (1.03–1.34)*	6.34 (−0.77–13.46)
SM by VC		1.10 (0.97–1.24)	6.11 (0.29–11.93)*
**Interactions (ACEs = 0)**			
SM by SH		0.97 (0.80–1.18)	−4.11 (−15.04–6.81)
SH by VC		0.96 (0.78–1.17)	−3.63 (−14.74–7.49)
SM by VC		0.98 (0.81–1.18)	−2.47 (−18.68–13.75)
**Interactions (ACEs ≥ 1)**			
SM by SH		1.27 (1.09–1.49)^**^	6.09 (0.35–11.83)*
SH by VC		1.26 (1.08–1.47)^**^	6.71 (−0.30–13.73)
SM by VC		1.18 (1.02–1.37)*	1.99 (−7.54–11.51)
**Interactions (ACEs ≥ 1 and adjusted for adult trauma)**			
SM by SH		1.27 (1.09–1.49)^**^	10.23 (−8.14–28.60)
SH by VC		1.26 (1.07–0.47)^**^	17.67 (−13.52–48.85)
SM by VC		1.18 (1.01–1.37)*	7.63 (−23.37–38.65)
**Interactions (adult trauma = 0)**			
SM by SH		1.12 (0.87–1.45)	−2.27 (−14.27–9.72)
SH by VC		1.09 (0.84–1.41)	−2.30 (−15.34–10.75)
SM by VC		1.02 (0.79–1.32)	−8.28 (−31.99–15.44)
**Interactions (adult trauma ≥ 1)**			
SM by SH		1.28 (1.09–1.51)^**^	5.50 (−0.49–11.49)
SH by VC		1.27 (1.08–1.49)^**^	6.34 (−0.77–13.46)
SM by VC		1.18 (1.01–1.37)*	6.11 (0.29–11.93)*
**Interactions (adult trauma ≥ 1 and adjusted for ACEs)**			
SM by SH		1.25 (1.07–1.47)^**^	14.35 (−10.97–39.76)
SH by VC		1.23 (1.05–1.45)*	23.97 (−17.48–65.42)
SM by VC		1.15 (0.99–1.34)	−2.73 (−7.56–2.09)

We found interactions between SM and SH and SH and VC, on the multiplicative scale; and between SM and VC on the additive scale. We then divided participants according to whether they reported experiencing ACEs and also adult trauma. There were no interactions between the three syndemic components among men who did not report ACEs. For those who reported ACEs, the table shows that ORs increased and there was an additional interaction between SM and VC on the multiplicative scale; there was an interaction between SM and SH on the additive scale.

We next divided participants according to whether they had experienced adult trauma. There were no interactions between syndemic components among those reporting no adult trauma. For those who reported trauma, ORs showed an increase, and there was an additional interaction observed between SM and VC on the multiplicative scale; there was an interaction between SM and VC observed on the additive scale.

## Model of serially causal epidemics

**Figure 1.**
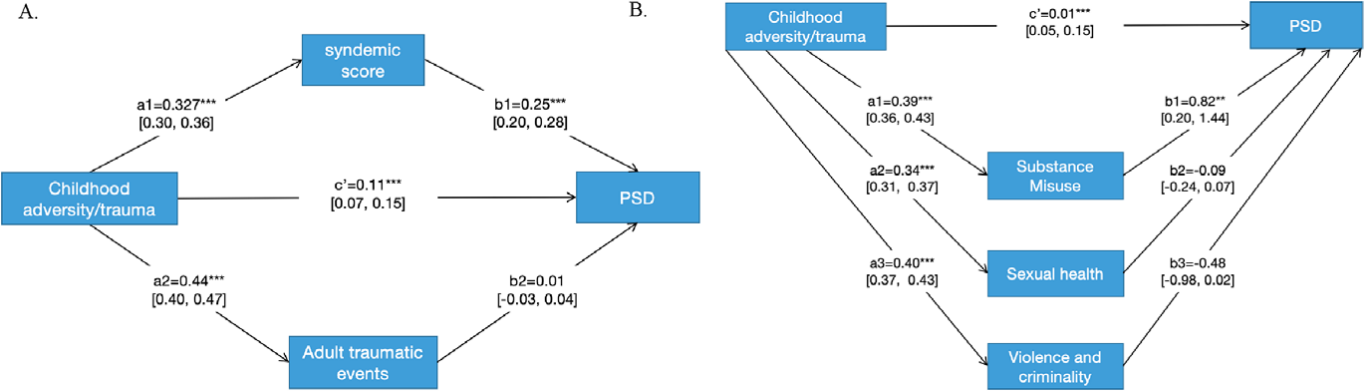
Testing the model of serially causal epidemics using mediation analysis on PSD. (a) For syndemic scores and traumatic events. (b) For disaggregated components of the syndemic.

Figure 1a tests the model of serially causal epidemics using mediation analysis. The model shows good fit: Apha = 1, rhoC = 1, AVE = 1 (for good fit Alpha, rhoC, and rhoA should exceed 0.7, and AVE exceed 0.5). We estimated statistically significant direct effects of CA on PSD. The direct effects of CA on syndemic score and adult trauma were also statistically significant. The indirect effect of syndemic score on PSD was also significant but not the indirect effect of adult trauma. Using bootstrapping, we found that the combined mediated effect of the syndemic and adult trauma was 42.4% of the total effect of CA on PSD. Adult traumatic events alone mediated only 1.4% of the effect compared to 41.0% mediated by the syndemic.

Figure 1b shows the disaggregated mediation effects of SM, SH, and VC on PSD, excluding adult traumatic events. The model showed good fit: Apha = 1, rhoC = 1, AVE = 1. The indirect effect of SM was significant but those of SH and VC were not significant. Because SM, SH, and VC demonstrated interactions, the percentage mediated by SM could not be estimated for clinical purposes relative to the direct effects and totaled >100% (208.3%) in the model.

## Model of mutually causal epidemics

**Figure 2.**
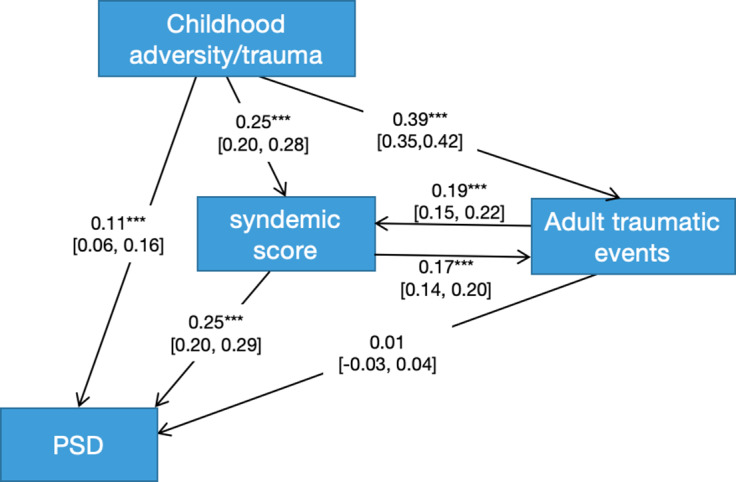
Testing the model of mutually causal epidemics using path analysis on PSD.

Figure 2 shows the model of mutually causal epidemics using path analysis. The model showed good fit: Alpha = 1, rho C = 1, AVE = 1. CA showed a significant association with PSD along a direct pathway for a subgroup of participants. CA showed two additional pathways with larger statistical associations with both syndemic scores and number of adult traumatic events. There were significant bi-directional associations between syndemic scores and adult traumatic events. There was a significant association on a pathway between syndemic scores and PSD, which showed a stronger association than that observed along the direct pathway. However, the association between adult trauma and PSD was not significant.

## Contextual factors, PSD, and two PSD psychosis subtypes

**Figure 3.**
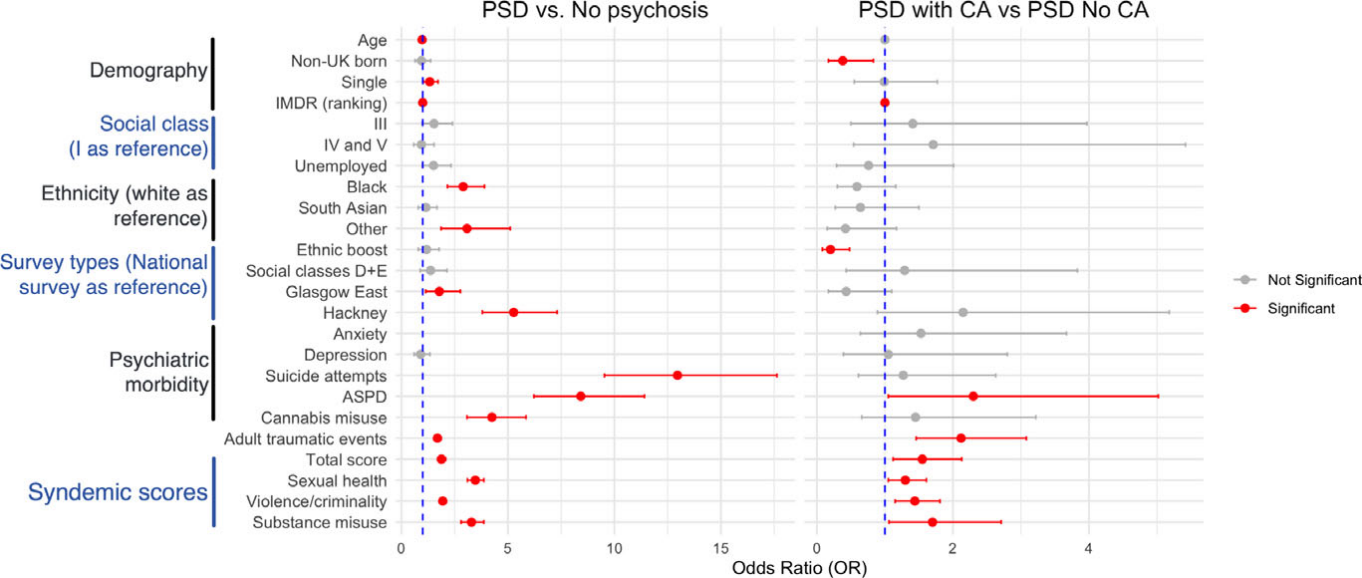
Summary comparison of contextual demographics factors, psychopathology and syndemic components according to reported child adversity.

Figure 3 and Table S3 compare contextual demographic factors, psychopathology, and syndemic components according to PSD (all cases) and according to whether or not PSD had been preceded by child adversity. PSD was more prevalent among single men, black and other ethnic subgroups, younger men, and those residing in somewhat more socioeconomically deprived areas. When comparing demography according to CA, men with PSD and reporting CA were more likely to be UK born, residing in more socioeconomically deprived areas. Men in the Glasgow East and Hackney samples showed a significantly higher prevalence of PSD.

Participants with PSD showed substantial differences for co-occurring suicide attempts and ASPD but not depression. Comparing PSD subgroups according to the presence or absence of CA, no differences were found in either psychosis measures or co-occurring conditions, except that ASPD was more common among those reporting CA.

Total syndemic scores and disaggregated scores for SH, VC, and adult traumatic events were significantly higher among men with PSD. These were all significantly higher among men with PSD who reported CA.

## Discussion

Our previous work established important findings on the role of syndemics in psychosis and ethnic disparities (Coid et al., 2020) and the role of childhood adversity in shaping these syndemic effects (Zhang & Coid, 2024). This study takes the next step by integrating multiple syndemic models and considers additional factors, including traumatic adult life events. By applying more sophisticated analytical techniques such as path modeling and mediation analysis, our study provides deeper insights into the potentially causal pathways that lead to PSD and indicate potential population-level interventions. Our findings also support a broad division of PSD into two groups: (i) PSD cases due to social determinants, and (ii) cases presumed due to biological/inherent causes and suggest a preventive strategy for PSD due to social determinants. Approximately two-thirds of men in our study had one or more experiences of CA. CA was the key social determinant of a future pathway involving the syndemic showing multiplicative effects of SM, SH, and VC, which ultimately led to PSD. CA also increased the risk of traumatic events in adulthood, as previously observed (Butler, Quigg, & Bellis, 2020, Fereidooni et al., 2023, Walker, Freud, Ellis, Fraine, & Wilson, 2019, Walker & Wamser-Nanney, 2023). We found adult traumatic events showed bidirectional effects, with the syndemic impacting on traumatic events and traumatic events impacting on the syndemic. Traumatic events ultimately increased risk of PSD. However, this was along the syndemic pathway and traumatic events had no direct effects. Previous research has not found strong evidence of adult trauma leading to psychosis (Beards et al., 2013).

Clinical symptomatic presentations of PSD were the same for individual cases despite our division according to social determinants or biological/inherent causes. This is important because cases cannot be accurately differentiated based on a clinical assessment of psychotic symptoms alone. Future research should investigate whether underlying neuropathology differs according to this new classification and if there are differing responses to treatments, such as trauma-based therapies (Reid, Cole, Malik, Bell, & Bloomfield, 2024).

## Testing three models: implications for population interventions

The model of mutually causal epidemics (Chakrapani et al., 2019) received partial support from our path analysis. The syndemic showed a significant association with adult traumatic events, and adult traumatic events with the syndemic. Ideally, when testing a model of mutually causal epidemics, the exposures should be considered causative of each other. It could be argued that behaviors involving VC, SH, and SM cause traumatic events of relationship breakdown, loss of employment, homelessness, etc. and vice versa. However, because there is a clear temporal relationship between CA and subsequent factors in adulthood, our findings using path analysis seemed more appropriate to the model of serially causal epidemics than the mutually causal model: an accumulation of stressors in childhood lead to development of both syndemic factors and traumatic life events which then interact. These, in turn, ‘snowball’ (Ferlatte, Dulai, Hottes, Trussler, & Marchand, 2015) to increase the risk of PSD. This further reinforces a core preventive strategy of targeting the exposures occurring earlier in childhood, aimed to reduce later risk by preventing the subsequent cascades of risk factors.

Mediation analysis and path analysis further emphasized that targeting exposures of CA at the population level is most important in preventing subsequent cascades of the multiple psychosocial problems preceding PSD. We observed a direct relationship between CA and PSD in some cases. This may constitute a different underlying etiological pathway from that involving the syndemic, possibly acting through individual CA factors having greater impact, such as childhood sexual abuse (Briggs, Amaya-Jackson, Putnam, & Putnam, 2021). Alternatively, through additive effects of multiple factors, for which there is stronger evidence (Petruccelli, Davis, & Berman, 2019). It could also involve gene × environment interactions which requires further investigation. We also found that effects of CA on PSD were substantially mediated by the syndemic but not by adult traumatic events. However, when testing mediating effects of individual components of the syndemic, neither VC nor SH were mediators, with mediation due to SM. This finding supports a single component intervention strategy as effective in mitigating the impact of SM rather than a multi-component additionally incorporating VC and SH.

Single-component interventions are indicated in situations of budgetary constraints, limited resources, or where there are no known health-care interventions that are effective for one or more components of a syndemic. It could be argued that healthcare interventions are not widely effective for VC. Tsai’s (2018) model of synergistically interacting epidemics implies that a single component intervention designed to eliminate a single component of the syndemic will reduce the risk of PSD to a greater degree than would be expected if no interactions were present. However, our findings suggest that the decision on which component of the syndemic to eliminate largely depends on the other two models we tested rather than relying solely on demonstrating synergistic interactions. Support for a single component intervention involving elimination or reduction of SM was supported. Similarly, preventing or mitigating impact of traumatic adult events would not be an effective single component intervention. In summary, strategies mitigating these subsidiary factors, such as VC and SH, are still likely to be beneficial because they exacerbate SM and should therefore be considered. However, the core strategy in childhood would be targeted at preventing CA and in adolescence/early adulthood aimed at reducing SM, particularly among individuals who have previously experienced CA and are thereby at higher risk of PSD, possibly through altered brain development.

## Population interventions

Our study offers new perspectives for prevention and intervention. We found that syndemic interactions were largely conditional on experiencing child adversity. Although there were few additive interactions, these were important because the two exposures of SH and SM interacting additively indicated that by focusing on addressing the cumulative effect of both exposures simultaneously in a single intervention would be likely to lead to a more effective intervention to reduce the risk of PSD compared to targeting both in isolation in two or more separate interventions. This would at first appear to contradict the findings of the effects of CA on PSD being mediated by SM alone. However, the practical significance of these findings means that although a single intervention targeting both SM and SH simultaneously would be more likely to have a greater impact, the key risk factor for PSD along the syndemic pathway from CA was SM. A single-component intervention targeting SM would still be effective and may ultimately be more practical than attempting to target both SM and SH or SM and VC.

Because CA and syndemic factors were substantial contributors to PSD, population interventions to mitigate their effects should ideally be targeted among subgroups where these effects are concentrated, where the overall prevalence of PSD is high, and ideally where PSD specifically due to social determinants is most prevalent. We found, as expected, that men with PSD were younger, more likely to be single, residing in areas of higher socioeconomic deprivation, more likely to be black and from other minority subgroups, corresponding to epidemiological studies of clinical psychosis (Halvorsrud, Nazroo, Otis, Brown Hajdukova, & Bhui, 2019). PSD associated with social determinants was more prevalent in the most socioeconomically deprived areas and less prevalent both among non-UK-born immigrants and in our nationally representative ethnic minority boost sample, suggesting that preventive interventions cannot simply be determined by demographic characteristics and that populations with high levels of CA and syndemic scores should be targeted.

Associations with ethnicity were highly complex and influenced by differing area-level effects. The overall high prevalence of PSD in Hackney, particularly among black men, appeared to be influenced by the high level of PSD due to social determinants in that location. However, the representative national boost sample also showed a higher prevalence of PSD among black men, but this was due to biological/inherent causes. This new finding requires further investigation as it suggests that overall higher rates observed in black men in the UK are due to two processes impacting simultaneously (both social determinants and biological/inherent causes) with both having greater impact on black men than other ethnic groups.

Implementing population-level public health interventions in socioeconomically deprived areas and areas with high levels of ethnic minorities presents several ethical considerations and needs to avoid exacerbating existing inequalities, stigmatization, or unintended negative consequences. These populations may be less empowered to make informed decisions about their health and interventions focused disproportionately on specific groups risk oversimplifying root causes of disparities, may not address broader contextual issues, such as racism and poverty. Focusing on child adversity or substance misuse in these populations may lead to perceptions they are inherently more ‘problematic’, reinforcing negative stereotypes, deepening existing social divisions, and marginalization. Programs may frame these groups as ‘victims’ in need of external intervention, rather than recognizing resilience and capacity for self-determination. Public health interventions in socioeconomically disadvantaged or ethnically marginalized areas can inadvertently lead to over-policing and even social unrest, especially if interventions are perceived as surveillance. To overcome these problems, culturally competent and community-centered approaches are crucial, involving communities in the design and implementation. Focusing on broader social determinants of health (e.g. housing, education, income inequality) alongside the specific health interventions we propose, can address the root causes of health disparities without focusing solely on individual behaviors. Finally, when addressing child adversity and substance misuse, adopting a trauma-informed approach can help prevent blaming individuals for their circumstances.

## Limitations

The most important limitation of our study is the use of cross-sectional data limited to self-report instruments to investigate models which are intended to test causation. This means that no causal inferences can ultimately be made. Our findings must therefore be replicated in the future using longitudinal data to confirm the temporal relationships between the identified factors and PSD outcomes. This means that our proposed preventive strategies are based on observed associations rather than causal evidence and emphasize the need for further validation in future studies. A further limitation is the absence of biomarkers. Classification of cases into those due to biological/inherent causes is based on the *absence* of social factors and limited to those measured in our surveys. For example, unmeasured gene × environment interactions may have contributed and could be underlying explanations for direct pathways such as from CA to PSD. We did not have an accurate measure of the clinical status of our participants with PSD to determine whether they had experienced life-time psychotic illness or were high-risk cases. However, we used a method that has previously indicated a substantial and clinically significant probability for psychotic illness found among male prisoners (Coid & Ullrich, 2011).

Our surveys were restricted to adult men. Previous studies have observed similar risk factors to the components of the syndemic we described among women, particularly those experiencing intimate partner violence at risk of HIV (Singer, 2009). The RLS method does not provide detailed information on the number of men who declined to participate. However, it is based on the national census, and participants who decline are replaced by another who fits the same demographic characteristics identified from the same small area (see supplementary file). Finally, we did not use a standardized instrument to identify ACEs. However, these are still under development. Our measures are likely to have been robust and covered the more severe traumatic experiences of childhood rather than inferring trauma on the basis of questions regarding parents with mental illness and SM, as included in some available instruments.

Our dataset’s inclusion of and focus on men from areas of high socioeconomic deprivation, such as Hackney and Glasgow East, limits the generalizability of our findings to broader or more diverse populations. Our observed syndemic interactions may also not fully apply to women, individuals from rural areas, or populations outside the UK. This means that while our findings provide insights into the syndemic processes within the studied subgroup, population-level interventions should be designed with caution, ensuring they are tailored to the specific needs of diverse populations.

## Implications

Our findings confirmed a broad category of PSD due to social determinants and involving differing pathways from CA for testing in future longitudinal research. We propose a hypothetical mechanism in which CA leads to developmental brain abnormalities but also increases the risk of multiple adverse risk factors during childhood and adolescence, including high-risk behaviors, such as SM, which can in turn trigger PSD. We found direct effects of CA on PSD but stronger effects along a second pathway where CA contributed directly to a cascade of risk factors including VC, SH, and SM, which were specific to PSD (Zhang & Coid, 2024). Our findings suggest that prevention of CA should be a primary public mental health preventive strategy but that a single component strategy in adolescence/early adulthood targeting SM is also likely to be effective.

Further research is required to identify contextual factors that determine whether interventions should be targeted on specific population groups, such as those experiencing syndemics which are particularly at risk, or can potentially involve the entire population (Frohlich & Potvin, 2008; Rose, 2001; Aagaard-Hansen, Hindhede, & Terkildsen Maindal, 2023). However, if syndemics are localized and do not extend to entire populations (Singer, Bulled, & Leatherman, 2022), new methods need to be developed to identify which populations require public health interventions, together with the contextual factors leading to the multimorbidity in which syndemics occur. Other syndemic factors need to be explored, including economic instability and access to mental health services, and testing these models in diverse populations, including among women and in non-urban populations. More information is needed on why some populations experiencing adverse contextual factors experience syndemics while others experiencing the same factors do not, and the implications for prevention.

## Supporting information

Zhang and Coid supplementary material Zhang and Coid supplementary material

## Supplementary material

The supplementary material for this article can be found at http://doi.org/10.1017/S0033291725000455.
