# Supplementary material for: Testing syndemic models along pathways to psychotic spectrum disorder: implications for population-level preventive interventions
Source: Psychol Med. 2025 Mar 13;55:e85. doi: 10.1017/S0033291725000455 (PMC12080663; doi:10.1017/S0033291725000455)
Supplement: Zhang and Coid supplementary material [file S0033291725000455sup001.doc]

**Supplementary material**

**Testing syndemic models along pathways to psychotic spectrum disorder: Implications for population-level interventions.**

**Contents**

1. Table S1 Demographic characteristics, psychosis, and childhood adversity in the national and additional surveys (n=7536)
2. Table S2 Adjusted associations of psychiatric morbidity, substance misuse, sexual health, violence and criminality, childhood adversity, and traumatic adult experiences with PSD (n=7461)
3. Table S3 Comparison of contextual demographic factors, psychopathology, and syndemic components according to reported child adversity (n=7461)
4. Why were health-related behaviours of substance misuse, risky sexual behaviour, and violence selected for testing syndemic interactions with psychotic symptoms as the outcome?
5. The First Men’s Modern Lifestyles Survey.
6. The Second Men’s Modern Lifestyles Survey.
7. Quota Sampling and the 2011 Young Men's Health Survey - Report from ICM (Surveying Company).

**Table S1 Demographic characteristics, psychosis, and childhood adversity in the national and additional surveys (n=7536^§^)**

|  | **Main survey (n=3247 43.5%)** | **BME boost (n=1540 20.6%)** | **Lower social classes (n=1002 13.4%)** | **London, Hackney (n=883 11.8%)** | **Glasgow East (n=789 10.5%)** | **F/χ^2^** |
| --- | --- | --- | --- | --- | --- | --- |
| Non-UK born | 285 (8.9%) | 495 (33.4%) | 73 (7.4%) | 215 (25.8%) | 12 (1.5%) | 722.67^***^ |
| Single | 1374 (42.7%) | 783 (52.7%) | 457 (45.8%) | 501 (57.1%) | 563 (71.6%) | 161.38^***^ |
| **Social class** |  |  |  |  |  | 522.92^***^ |
| I and II | 558 (17.2%) | 223 (14.5%) | 19 (1.9%) | 180 (20.4%) | 39 (4.9%) |  |
| III | 982 (30.2%) | 373 (24.2%) | 115 (11.5%) | 255 (28.9%) | 174 (22.1%) |  |
| IV and V | 717 (22.1%) | 405 (26.3%) | 393 (39.2%) | 200 (22.7%) | 163 (20.7%) |  |
| Unemployed | 990 (30.5%) | 539 (35%) | 475 (47.4%) | 248 (28.1%) | 412 (52.3%) |  |
| **Ethnicity** |  |  |  |  |  | 4724.66^***^ |
| White | 2900 (89.6%) | 0 | 856 (86%) | 284 (32.3%) | 775 (98.6%) |  |
| Black | 137 (4.2%) | 589 (62.1%) | 75 (7.5%) | 287 (32.6%) | 4 (0.5%) |  |
| South Asian | 148 (4.6%) | 816 (49.9%) | 56 (5.6%) | 246 (28%) | 6 (0.8%) |  |
| Other | 51 (1.6%) | 132 (8.6%) | 8 (0.8%) | 63 (7.2%) | 1 (0.1%) |  |
|  | **mean±SD** | **mean±SD** | **mean±SD** | **mean±SD** | **mean±SD** |  |
| Age | 34.16±16.05 | 31.26±34.51 | 33.15±15.05 | 27.72±7.91 | 26.78±3.19 | 243.20^***^ |
| IMDR (ranking) | 12455.69±10746.66 | 6639.67±8659.8 | 6238.47±5746.74 | 3603.88±2401.1 | 1292.08±1542.98 | 1830.00^***^ |
| General syndemic score^#^ | -0.05±1.06 | -0.24±1.45 | 0.01±1.14 | 0.68±1.77 | 0.15±1.4 | 64.65^***^ |
| Mean no. ACEs | 0.56±0.96 | 0.33±1.05 | 0.64±1.08 | 0.58±0.99 | 0.67±0.98 | 14.73^***^ |
| Mean no. adult trauma | 0.67±1.1 | 0.38±1.24 | 0.77±1.22 | 0.48±0.88 | 0.78±1.17 | 0.87 |
| PSQ scores | 0.2±0.61 | 0.24±1.05 | 0.27±0.72 | 0.49±1.08 | 0.25±0.79 | 33.85^***^ |
|  | **n (%)** | **n (%)** | **n (%)** | **n (%)** | **n (%)** |  |
| Psychosis (latent class) | 75 (2.2%) | 36 (2.4%) | 30 (2.9%) | 86 (9.7%) | 30 (3.8%) | 126.92^***^ |

**^§^** Weights were constructed for each survey using Random Iterative Method (RIM) weighting to ensure representativity of the sample.

**^#^**available for 4618 men with no missing value for variables used to calculate general syndemic score.

**Table S2 Adjusted associations of psychiatric morbidity, substance misuse, sexual health, violence and criminality, childhood adversity, and traumatic adult experiences with PSD (n=7461)**

|  | **Other men** | | **PSD (latent class)** | |
| --- | --- | --- | --- | --- |
|  | **N=7212 (96.7%)** | | **N=249 (3.3%)** | |
|  | **N (%)** | **OR** | **N (%)** | **OR (95%CI)** |
| **Comorbidity** |  |  |  |  |
| Anxiety | 894 (12.8%) | ref (1) | 198 (79.8%) | 30.85 (21.58-44.11)^***^ |
| Depression | 764 (11%) | ref (1) | 35 (14.3%) | 0.9 (0.6-1.34) |
| Suicide attempts | 331 (4.8%) | ref (1) | 98 (40.3%) | 12.96 (9.53-17.63)^***^ |
| ASPD | 740 (11%) | ref (1) | 110 (49.1%) | 8.42 (6.22-11.41)^***^ |
| Alcohol dependence | 605 (8.8%) | ref (1) | 104 (42.8%) | 6.71 (4.98-9.05)^***^ |
| Drug dependence | 151 (2.2%) | ref (1) | 52 (21.6%) | 9.09 (6.16-13.4)^***^ |
| Cannabis misuse | 620 (8.6%) | ref (1) | 70 (28.1%) | 4.25 (3.08-5.85)^***^ |
| **Sexual health** |  |  |  |  |
| STD | 568 (8.9%) | ref (1) | 67 (29%) | 3.7 (2.68-5.11)^***^ |
| Sex worker (ever) | 234 (3.7%) | ref (1) | 41 (17.7%) | 4.05 (2.71-6.05)^***^ |
| Anal sex | 1164 (18.4%) | ref (1) | 78 (33.8%) | 2.42 (1.79-3.28)^***^ |
| Coercive sex | 237 (4%) | ref (1) | 41 (18.7%) | 4.57 (3.05-6.84)^***^ |
| ≥10 sexual partners (past year) | 384 (6.3%) | ref (1) | 57 (24.9%) | 3.48 (2.43-4.98)^***^ |
| Rare contraceptive use | 2031 (34.2%) | ref (1) | 101 (45.9%) | 1.83 (1.37-2.45)^***^ |
| MSM | 260 (4%) | ref (1) | 30 (13%) | 2.46 (1.56-3.87)^***^ |
| **Violence and criminality** |  |  |  |  |
| Repeated violence | 1104 (16.1%) | ref (1) | 110 (46.6%) | 4.26 (3.17-5.72)^***^ |
| IPV | 589 (8.7%) | ref (1) | 78 (34.5%) | 5.97 (4.35-8.19)^***^ |
| Fear violent victimization | 1149 (17.5%) | ref (1) | 122 (53.5%) | 4.87 (3.64-6.5)^***^ |
| Carried weapon | 388 (5.8%) | ref (1) | 68 (29.7%) | 5.65 (4.07-7.84)^***^ |
| Gang membership | 87 (1.3%) | ref (1) | 45 (20.1%) | 12.9 (8.25-20.17)^***^ |
| Peers encourage crime | 763 (10.7%) | ref (1) | 66 (26.9%) | 4.2 (3.03-5.82)^***^ |
| Ever in prison | 318 (4.4%) | ref (1) | 43 (17.4%) | 5.33 (3.62-7.85)^***^ |
| **Childhood adversity** |  |  |  |  |
| Bullying | 1734 (24%) | ref (1) | 116 (46.6%) | 3.34 (2.53-4.4)^***^ |
| Violence in home | 699 (9.7%) | ref (1) | 76 (30.4%) | 4.28 (3.14-5.84)^***^ |
| Sexual abuse | 166 (2.3%) | ref (1) | 28 (11.2%) | 4.32 (2.71-6.88)^***^ |
| Physical abuse | 425 (5.9%) | ref (1) | 48 (19.3%) | 3.8 (2.65-5.43)^***^ |
| Neglect | 315 (4.4%) | ref (1) | 53 (21.2%) | 4.9 (3.44-6.99)^***^ |
| Serious illness/injury | 146 (2%) | ref (1) | 28 (11.2%) | 4.98 (3.08-8.07)^***^ |
| In care | 264 (3.8%) | ref (1) | 38 (16.6%) | 4.37 (2.92-6.53)^***^ |
| no ACEs | 4603 (66.8%) | ref (1) | 67 (29.3%) | 0.18 (0.13-0.25)^***^ |
| >=2 ACEs | 756 (11%) | ref (1) | 95 (41.5%) | 5.62 (4.17-7.58)^***^ |
| **Adult traumatic life events** | |  |  |  |
| Domestic violence | 203 (2.8%) | ref (1) | 30 (12%) | 4.88 (3.12-7.64)^***^ |
| Sexual assault | 67 (0.9%) | ref (1) | 20 (8%) | 6.92 (3.90-12.28)^***^ |
| Life threatening injury | 183 (2.5%) | ref (1) | 29 (11.6%) | 4.6 (2.88-7.33)^***^ |
| Separation/divorce | 728 (10.1%) | ref (1) | 36 (14.5%) | 1.67 (1.1-2.53)^*^ |
| Fired from job | 1352 (18.7%) | ref (1) | 52 (20.9%) | 1.35 (0.95-1.92) |
| Homelessness | 502 (7%) | ref (1) | 54 (21.7%) | 4.85 (3.43-6.87)^***^ |
| Serious money problems | 1206 (16.7%) | ref (1) | 96 (38.4%) | 3.27 (2.44-4.37)^***^ |
| No events/victimization | 4765 (66.1%) | ref (1) | 91 (36.5%) | 0.24 (0.18-0.32)^***^ |
| >=2 events/victimization | 1139 (15.8%) | ref (1) | 87 (34.9%) | 3.42 (2.52-4.65)^***^ |

Adjusted for age, non-UK born, single, ethnicity, social class, IMDR (score), and survey type

**Table S3 Comparison of contextual demographic factors, psychopathology, and syndemic components according to reported child adversity (n=7461)**

|  | **No psychosis**  **n=7212 (96.7%)** | **PSD**  **n=249 (3.3%)** | **OR (95% CI)** | **PSD without childhood adversity  n=67 (29.3%)** | **PSD with childhood adversity n=162 (70.7%)** | **OR (95% CI)** |
| --- | --- | --- | --- | --- | --- | --- |
| **Demography** | **n (%)** | **n (%)** |  | **n (%)** | **n (%)** |  |
| Non-UK born | 1049 (14.8%) | 32 (13.9%) | 0.94 (0.64-1.37) | 14 (24.6%) | 17 (11.0%) | 0.38 (0.17-0.83)^*^ |
| Single | 3538 (49.7%) | 141 (56.9%) | 1.33 (1.03-1.72)^*^ | 38 (58.5%) | 94 (58.0%) | 0.99 (0.55-1.77) |
| **Social class** |  |  |  |  |  |  |
| I and II | 992 (13.8%) | 26 (10.4%) | ref (1) | 7 (10.4%) | 16 (9.9%) | ref (1) |
| III | 1826 (25.3%) | 74 (29.7%) | 1.53 (0.97-2.4) | 16 (23.9%) | 53 (32.7%) | 1.41 (0.5-3.97) |
| IV and V | 1833 (25.4%) | 46 (18.5%) | 0.94 (0.58-1.53) | 9 (13.4%) | 33 (20.4%) | 1.71 (0.54-5.42) |
| Unemployed | 2561 (35.5%) | 103 (41.4%) | 1.51 (0.98-2.33) | 35 (52.2%) | 60 (37%) | 0.76 (0.29-2.01) |
| **Ethnicity** |  |  |  |  |  |  |
| White | 4696 (65.3%) | 120 (48%) | ref (1) | 27 (40.9%) | 89 (54.9%) | ref (1) |
| Black | 1017 (14.2%) | 75 (30.0%) | 2.9 (2.16-3.9)^***^ | 22 (33.3%) | 41 (25.3%) | 0.59 (0.3-1.16) |
| South Asian | 1236 (17.2%) | 36 (14.4%) | 1.15 (0.79-1.67) | 10 (15.2%) | 22 (13.6%) | 0.64 (0.27-1.5) |
| Other | 238 (3.3%) | 19 (7.6%) | 3.08 (1.86-5.11)^***^ | 7 (10.6%) | 10 (6.2%) | 0.42 (0.15-1.17) |
|  | **mean±SD** | **mean±SD** |  | **mean±SD** | **mean±SD** |  |
| Age | 32.02±27.98 | 20.20±17.04 | 0.97 (0.96-0.99)^***^ | 29.3±23.37 | 27.41±14.79 | 1 (0.97-1.04) |
| IMDR (ranking) | 8266.29±9412.64 | 6137.50±7348.65 | 1.00 (1.00-1.00)^***^ | 6490.97±9030.62 | 6107.11±6635.88 | 1.00 (1.00-1.00)^***^ |
| **Survey types** |  |  |  |  |  |  |
| National survey | 3180 (97.9%) | 67 (2.1%) | Ref (1) | 16 (25.0%) | 48 (75.0%) | Ref (1) |
| Ethnic boost | 1503 (97.6%) | 37 (2.4%) | 1.18 (0.79-1.77) | 23 (62.2%) | 14 (37.8%) | 0.20 (0.08-0.48)^***^ |
| Social classes D+E | 974 (97.2%) | 28 (2.8%) | 1.37 (0.88-2.14) | 6 (21.4%) | 22 (78.6%) | 1.29 (0.43-3.83) |
| Glasgow East | 795 (90%) | 88 (10%) | 1.78 (1.14-2.77)^*^ | 10 (13.7%) | 63 (86.3%) | 0.43 (0.17-1.1) |
| Hackney | 761 (96.5%) | 28 (3.5%) | 5.27 (3.8-7.31)^***^ | 12 (42.9%) | 16 (57.1%) | 2.15 (0.89-5.18) |
| **Psychiatric morbidity** | **n (%)** | **n (%)** |  | **n (%)** | **n (%)** |  |
| Psychosis (PSQ>=3) |  |  |  | 47 (69.1%) | 99 (59.3%) | 0.62 (0.30-1.29) |
| PSQ hypomania |  |  |  | 28 (41.8%) | 68 (42%) | 0.98 (0.48-2.03) |
| PSQ thought insertion |  |  |  | 30 (45.5%) | 71 (43.8%) | 0.93 (0.46-1.86) |
| PSQ paranoid |  |  |  | 50 (74.6%) | 129 (79.6%) | 1.96 (0.85-4.55) |
| PSQ strange experience |  |  |  | 57 (85.1%) | 139 (85.8%) | 1.39 (0.55-3.49) |
| PSQ hallucination |  |  |  | 43 (65.2%) | 75 (46%) | 0.50 (0.24-1.04) |
| Anxiety |  |  |  | 49 (73.1%) | 135 (83.9%) | 1.53 (0.64-3.67) |
| Depression | 773 (11%) | 37 (14.6%) | 0.9 (0.60-1.34) | 8 (11.9%) | 24 (15.1%) | 1.05 (0.39-2.80) |
| Suicide attempts | 334 (4.8%) | 103 (41.2%) | 12.96 (9.53-17.63)^***^ | 19 (28.4%) | 71 (44.7%) | 1.27 (0.61-2.63) |
| ASPD | 744 (11%) | 113 (48.9%) | 8.42 (6.22-11.41)^***^ | 16 (26.2%) | 83 (57.2%) | 2.30 (1.05-5.02)^*^ |
|  | **mean±SD** | **mean±SD** |  | **mean±SD** | **mean±SD** |  |
| Syndemic score | -0.05±1.79 | 1.21±1.89 | 1.88 (1.7-2.08)^***^ | 0.87±1.62 | 1.92±1.88 | 1.55 (1.12-2.13)^**^ |
| Sexual health | 0.54±1.15 | 5.18±2.74 | 3.47 (3.1-3.87)^***^ | 4.3±2.75 | 5±2.23 | 1.3 (1.05-1.61)^*^ |
| Violence/criminality | 0.61±1.10 | 2.05±1.94 | 1.94 (1.79-2.11)^***^ | 1.26±1.43 | 2.29±2.02 | 1.44 (1.15-1.81)^**^ |
| Adult traumatic events | 0.59±1.12 | 1.22±1.51 | 1.69 (1.53-1.87)^***^ | 0.67±1.77 | 1.5±1.39 | 2.12 (1.46-3.08)^***^ |

Adjusted for age, non-UK born, single, ethnicity, social class, IMDR (score), and survey type for analysis of psychiatric morbidity.

**Why were health-related behaviours of substance misuse, risky sexual behaviour, and violence selected for testing syndemic interactions with psychotic symptoms as the outcome?**

**Background**

The selection of substance misuse, risky sexual behaviour, and violence/criminality will come as no surprise to academics acquainted with the work of Singer^[1]^. Singer first defined a syndemic in the context of his work on the AIDS crisis among the urban poor, describing the SAVA epidemic^[2]^. This work had been preceded by that of Wallace and Fullilove who had studied inner-city decline in the USA during the 1980’s showing the explosion of violence, substance misuse, and AIDS in poor, minority inner-city populations in the USA, facing multiple political, economic and social challenges of unemployment, substandard housing or homelessness, poor nutrition, disrupted family and social relationships, population displacement and little or no access to healthcare^[3]^. Withdrawal of municipal funding due to tax cuts for the rich had led to fire departments being unfunded and multiple fires had resulted in population displacement from Harlem, New York, into the neighboring Bronx, further exacerbating these problems to constitute a public health crisis. Although not focusing on mental health, the authors observed that people from these areas had highest rates of admission to psychiatric hospitals in New York. The rate of HIV in Harlem at that time was the same as in sub-Saharan Africa. These studies described a concentration of diseases and health-related behaviours in minority populations but explained these within the context of political and other social forces in which these same populations had no influence or say. These experiences were described as structural violence, perpetrated by major institutions in society, referred to by Farmer^[4]^ as “a host of offenses against human dignity: extreme and relative poverty, social inequalities ranging from racism to gender inequality, and the more spectacular forms of violence that are human rights abuses”. Influenced by this theory, Singer^[1]^ described the term violence in this context as inequality of such gravity in amplifying human pain and suffering and so damaging as to constitute a form of sanctioned violence. For Singer, structural violence and violence often are found together because victims of structural violence themselves generate violence from their suffering. Daily living teaches that people experiencing structural violence have few options, an uncertain future, their lives hold little value in wider society, and they feel dispensable, with episodes of explosive anger, often found in their intimate relationships^[1]^.

Singer later refined these observations to show that diseases co-occur in particular temporal or geographical contexts because of harmful social conditions (disease concentration) and can interact at the level of populations and individuals, with mutually enhancing deleterious consequences (disease interaction) to constitute syndemics^[5-7]^. The overwhelming majority of published research in syndemics is in the HIV-related literature. Observations of substance misuse, risky sexual behaviour, and a violent lifestyle, both as perpetrator and victim, are frequent in these studies. Researchers are not considered to be “stigmatizing” their patients by reporting these findings. Academics and clinicians have negotiated with patients away from an early position where reporting risky sexual behaviour leading to an increased likelihood of positive HIV testing was considered “victim blaming” towards a wider recognition of public health risks endangering entire communities. However, the position remains very different for non-communicable psychiatric disorders where it is questioned whether health-related behaviours such as violence and risky sexual behaviour can have any impact on psychosis as outcome - although the position for substance misuse is different. To some extent, this concern regarding the findings of the present study reflects a misunderstanding of Singer’s key tenet that social forces are essential in the clustering of diseases in populations and that these are beyond the control of these populations at the individual level. Secondly, the relationship between these conditions and that they can be bi-directional.

Singer additionally pointed out that the impacts of structural violence on health are not always direct. Major inequalities in stress through adverse life circumstances, environmental conditions, diet, and self-destructive responses all act as mediators of disease effects. For our research, it became apparent that differences in levels of stress, but particularly differences in self-destructive responses secondary to that stress, profoundly influenced our selection of domains and variables that had not been tested in previous studies for their impact on psychosis. Singer listed examples of alcohol and drug abuse, obsessive gambling, and risky sexual behaviour as offering immediate relief but being ultimately self-destructive. Studies of the health status among the poor by physical location show that the sociophysical environment in which people live, including awareness of threats, danger, stress, discomfort, and alienation from their local social environment, are all critical determinants of their behaviour. Feelings of hopelessness and powerlessness in a community are good predictors of health and use of psychotropic drugs. These various strategies can be thought of as self-medication but reflect the lack of options in their lives, difficulties in self-regulation, and suffering. The long-term effects of these behaviours in situations of structural violence is ultimately to add to their suffering, stress, and psychopathology. An additional expression of structural violence is the ready availability of drugs among the poor, outlets selling alcohol, street dealers in illicit drugs, betting shops, and in some cases availability of weapons for sale such as knives and firearms, facilitated by mainstream social institutions and fiscally dominant social strata who are the beneficiaries.

**Context of Syndemic Research in UK**

The actual selection of the three domains of substance misuse, sexual behaviour, and violence/criminality in the first psychiatric study^[8]^ aimed to test a syndemic with psychosis as the outcome was initially pragmatic but profoundly influenced by the work of Singer^[1]^. A large research programme had been funded by the UK National Institute of Health Research into risk assessment for violence. One component was the identification of risk factors for violence at the population level. National datasets were already available from psychiatric morbidity surveys of the UK population and included women. However, further cross-sectional data were needed to study factors such as street gangs, ethnicity, social status, and violence in unusual populations known to have high rates of psychopathology, substance misuse, and multiple social problems which were thought to probably relate to these high rates of violence based on reviewing the literature on violence to develop questions for the survey. This study focused on men because rates of violence are higher among men but used a specific quota sampling method due to the difficulty in accessing hard-to-reach populations with high rates of violence and associated social problems. It was clear there was a lack of cross-sectional data on exceptional and unusual populations to compare with a general population reference group in the existing literature. As the research group became aware of the syndemic literature, it was also clear that by sampling from these areas we could additionally investigate syndemics,

The terms of funding gave considerable latitude to study design and two specific areas were selected for comparison with the general population: Glasgow East, Scotland, because it had gained notoriety in the past as having a high level of gang-related criminality and because it had been described in the media as “The most socioeconomically deprived area in western Europe.” Scotland also had the highest rates of homicide in Western Europe during the 1980’s, concentrated mainly in poor areas of Glasgow like Glasgow East. Gangs were no longer found to be of importance in Glasgow East by the time of the survey, but the level of substance misuse and dependence were exceptional for the UK, with Scotland by now having the highest rate of drug-related deaths in Europe and where the overall life expectancy remains shortest for the UK and Western Europe. Scotland currently has an “opioid crisis” equivalent to that of the USA. This became the subject of our second study on a syndemic with poor physical health leading to shortened life-expectancy as outcome of interactions between ongoing poor physical health, substance misuse, and psychiatric morbidity^[9]^. The other selected area was Hackney, east London, where the grant holder had worked as a clinician with in-patients in Forensic Psychiatry for over 20 years and had conducted an incidence study of psychosis^[10]^. This borough is similar to Lambeth in south London in having the highest proportion of people of African and Caribbean heritage in the UK, together with high numbers of persons originating from the Indian sub-continent in Hackney. It had been socioeconomically deprived for many years, although paradoxically the level of employment was higher than the UK in general due to the effect of being in London and by the time of the survey had begun a process of gentrification. More recently, official records showed it had the highest rate of new cases of psychotic illness registered with local psychiatric services in England in 2015. Our survey found that self-reported violence was high in Hackney among all ethnic groups and that 1 in 5 black men in the borough age 18-34 reported they were a member of a street gang^[11]^. Reporting these findings posed few problems at the time because they were new, did not constitute “a narrative these local communities did not want to hear”, and were not seen as “stigmatizing” at the time of publication of the paper which was the first in the psychiatric literature to report exceptionally high levels of psychopathology among gang members, including psychotic symptoms. However, this predated the later influence of critical justice theory on attitudes towards these areas of research.

For our research group, the data provided an opportunity to investigate whether the observation that high levels of crime and violence, repeatedly observed in the same urban areas showing both high prevalence and incidence rates of psychotic illness (particularly in the UK) since the first half of the 20th century were somehow related. Our first study suggested a new theoretical explanation for ethnic disparities in psychosis observed in UK inner-urban areas^[10, 12]^. It corresponded to research emphasising social determinants in the study of psychosis and urbanicity, indicating effects of social adversity and exclusion in relation to observed geographical variation. Although the study was primarily from the perspective of psychotic experiences as the primary outcome, bi-directionality with anxiety, drug and alcohol misuse, high-risk sexual behaviour, and violence and criminality should also be considered, given that each are potential outcomes and equally dependent on each other. The syndemic therefore corresponded to studies from the early 20th century onward, which have reported that these components co-occur and cluster geographically^[13, 14]^.

The key argument for selecting drug misuse, risky sexual behaviour and violence/criminality is that each of these health-related behaviours occur in populations undergoing stress and can themselves induce stress in individuals, corresponding to syndemic theory. They can be bidirectional with the outcome psychosis within populations experiencing structural violence. This corresponds to psychological theory in which symptoms such as paranoid thinking – part of the spectrum of psychosis – can be induced experimentally where this is mediated through anxiety^[15, 16]^.

**Drug misuse**

The choice of drug misuse in selection of variables should be beyond challenge. There are longstanding established nosological associations. For example, an earlier focus on alcohol and psychosis established alcoholic hallucinosis as an individual category of psychosis from the second half of the 19th century when psychiatrists started referring to alcohol-induced psychiatric disorder as a specific alcoholic psychosis, although this has declined in recent decades^[17]^. Amphetamine misuse can result in a psychosis indistinguishable from schizophrenia. Cocaine misuse and paranoid ideation are well-established and these variables are included in our study. Frequent cannabis users also have high risk of developing transitory psychotic symptoms and investigation studies consistently report an association between psychotic disorder and cannabis use^[18-20]^. Drug abuse is therefore a long-standing etiological factor for psychosis and a major environmental risk factor^[21, 22]^. In clinical practice, it is usually a function of severity of use and of addiction^[23]^. In terms of an association with psychosis as outcome it is usually assumed that substances have a direct effect. However, a broader view will include reverse causation, and lifestyle factors where becoming involved in the underground economy exerts extreme stress on individuals despite financial gain through criminal activity and which may be mediated by risk of becoming a victim of violence. Singer^[1]^ has argued that drug-addicted patients often have histories of difficulties with aggression, rage and depression and drug misuse often gives relief from dysphoric feelings of restlessness, anger, and rage^[24]^. Cameron and Jones^[25]^ have used the term “drugs of solace” to refer to providing solace, even in the short-term, from daily social stress, perceived failure, and damaged self-worth in the context of structural violence..

**High-risk sexual behaviour**

Risky sexual behaviour and self-rated poor mental health were found to be strongly correlated in a study of young persons, especially among females where it was associated with depression^[26]^. High-risk sexual behaviour is high among patients with severe mental disorders^[27]^ and it has been argued that mental illness leads to impaired judgement, impulsive sexual behaviour, sensitivity to personal rejection, and low self-esteem^[28]^. Park et al^[29]^ have described the effect of stress on compulsive sexual disorder, corresponding to Singer’s^[1]^ description of risky sexual behaviour in the context of structural violence. The behaviour can become compulsive and out of control, with individuals compulsively seeking out sexual activity despite bad consequences to themselves and others, ensuing problems in life, and ultimately deriving little pleasure. The behaviour often relieves anxiety. Stress levels have been shown to be positively correlated with compulsive sexual behaviour. Poor self-control is thought to mediate the relationship together with poor coping strategies. In individuals without coping strategies for stress there can be further discomfort and tension. Patients at risk of compulsive sexual behaviour can perform acts because they temporarily feel stable when they are satisfied in stressful situations. But the satisfaction from sexual behaviour can be strengthened to an addictive level^[30]^.

**Violence and Criminality**

Singer^[1]^ argues that “structural violence and interpersonal violence often go hand in hand, as victims of structural violence generate physical violence from their suffering.” Scandinavian case register studies and reviews show that violent convictions and arrests are higher among persons who receive diagnoses of schizophrenia and schizophrenia spectrum disorders. However, it was also concluded that most of the excess risk is mediated by substance abuse co-morbidity^[31]^. If this is considered more carefully it suggests that the excess of violence is substantially related to daily living factors in the lives pf psychotic patients rather than uniformly being due to symptoms of psychosis. In addition, these studies are highly limited by reliance on criminal justice statistics which miss the majority of violence, create bias where mentally disordered offenders are more likely to be processed by the courts and come to the attention of the police, and are using measures of the criminal processing process itself rather than actual violence. Nevertheless, case register studies emphasise the inter-relationship between substance misuse - violence/criminality - psychosis and their bidirectionally. These studies do not contain sufficient information to tell how many cases of violence were due to symptoms of psychosis being exacerbated by substance misuse and leading to violence. This is highly unlikely to be the sole explanation for these findings where lifestyle factors and social decline in the lives of persons with psychosis have resulted in becoming involved in violence, often after initially becoming victims themselves. Nevertheless, an interaction between violence and substance misuse was a key finding of our study. But from the perspective of structural violence theory, the notion of violence leading to violence through early childhood experience and persisting into adult life as both victim and perpetrator, together with the interactions between substance misuse and risky sexual behaviour in populations experiencing high levels of anxiety and stress are supportive of selection of these behaviours in our study. It should be added that our domain of violence/criminality also included fear of violent victimization.

**The First Men’s Modern Lifestyles Survey.**

The ‘Men’s Health and Modern Lifestyles Survey’ was carried out in 2009 by ICM for Queen Mary, University of London and was funded by The Maurice and Jacqueline Bennett Charitable Trust. The sample comprised 3025 men aged 18–64 years living in England, Wales and Scotland. A one-stage survey sought to interview a geodemographically representative sample of the male population of the United Kingdom through a random location methodological approach. Random location techniques utilize a full selection of geographic areas to be visited by interviewers, allied to quota sheets showing exactly who they must approach and interview within their target geography. This procedure necessitated the use of profiling statistics from the then most up-to-date Census (2001).

Within each Government Office Region, all output areas (OA) (averaging 150 households, and about which all demographic profiling information is known) were selected and listed in descending order of ACORN (A Classification Of Residential Neighbourhoods)^[32]^ type to place the most affluent OAs at the top of the list and the least affluent at the bottom. This applies a purely random variable into the selection of sampling locations. The total number of eligible male adults in each OA were then cumulated down the list. Using a random start and fixed sampling interval, the required number of OAs were selected. This process produces a sample of OAs with a probability of selection proportionate to size and was designed to produce a representative sample by ACORN type. A total of 250 OAs were selected, with interviewers required to achieve 12 interviews with eligible targets at each. All addresses that lay within selected OAs were potentially available for interview. With OA information cross-referenced against full address lists, interviewers were supplied with every single address that was eligible within each OA. A quota sheet was provided for each selected OA, which reflected the actual composition of eligible residents according to standard demographic criteria. These would include socio-demographic characteristics such as gender, ethnicity and working status (in addition to age). Interviewers were required to interview a sample profile that matched exactly that of the eligible OA population profile using the then up-to-date Office of National Statistics (ONS) population estimates information. This ensured that the sample was demographically representative at the micro-level, as well as geographically representative of males in the general population. If a participant refused to complete the questionnaire (approximately 23% of all participants approached), or was absent, another was located in the area with exactly the same demographic profile (age and social class) until the quota was filled.

The statistical reliability of this approach depended both on strictly defining the selection of the sampling points as well as in setting representative quotas at each point, and then meeting these quotas meticulously. Compliance with this procedure produced a fully representative data set. Self-report questionnaires were administered at home, with the respondent left to complete the questionnaire in their own time. The researcher either returned later that day or the next. Each questionnaire took approximately 45 minutes to complete. Participants were given £5 on completion of the questionnaire. A total of 3025 male adults completed the questionnaire. Study design and procedures were approved by the Queen Mary, University of London Human Research Ethics Committee.

**The Second Men’s Modern Lifestyles Survey.**

This study has been previously described briefly in a published paper^[11]^ and was part of a research programme into risk assessment of violence and identification of risk factors funded by UK National Institute of Health Research^[33]^. It was one component of the overall programme and was aimed to identify risk factors at the population level. In some cases, analyses were carried out combing data with national surveys of psychiatric morbidity that were available from the UK Department of Health^[33]^.

The survey was carried out in 2011 based on random location sampling and using the same method as the First Men’s Modern Lifestyles Survey. Individual sampling units (census areas of 150 households) were randomly selected within British regions in proportion to their population to derive a representative sample of young men (18–34 years) from England, Scotland and Wales. In this second survey, however, there were four additional, boost surveys over-sampling young Black and minority ethnic men, and those from lower social grades. Two boost surveys oversampled from output areas in locations characterised by high gang membership, violence, and social exclusion: the London Borough of Hackney and Glasgow East, Scotland. The same sampling principles applied to each survey type.

For the ethnic minority boost, sampling was carried out primarily in towns and cities based on a percentage cut-off, where it was known that BME persons would be located and that few would be found in villages and rural areas. The aim was to correspond to BME groups in the national UK survey (e.g. the proportion of men of South Asian heritage would be larger than black men to correspond to the national census findings). Each additional sample from special areas eg. Hackney, Glasgow East also corresponded according to ethnicity to the national census.

The self-administered questionnaire piloted in the previous survey was adapted and informed consent obtained from respondents, as before. Participants were contacted in person by interviewers and, if agreeing to participate, completed the pencil and paper questionnaire in private and returned it to the interviewer. All participants were paid £5 for taking part in the survey and all questionnaires were anonymised.

Weights were constructed for each survey using Random Iterative Method (RIM) weighting to ensure representativity of the sample. All descriptive and subsequent statistical comparisons were based on weighted data.

**Quota Sampling and the 2011 Young Men's Health Survey - Report from ICM (Surveying Company).**

Individuals were recruited by proportional quota sampling. This is a standard method that entails setting quotas for participants on a range of demographic factors and ensures that the sample interviewed is representative of the population of interest. It is particularly useful when investigating hard-to-reach samples, sensitive subjects such as violence, sexual behaviour, etc, and where it is anticipated that certain sub-sections of the population are less likely to complete interviews or questionnaires (e.g. young, male, lower social class, ethnic minorities, in areas of socioeconomic deprivation).

Quota sampling offers an alternative to probability sampling and is often used in market research and national surveys and becomes necessary if there is no listing of all those eligible to be included. It is more efficient as recruitment and sampling can be focused in areas in which the desired population are resident but does require good census data on the characteristics by which the quota are set. This method is preferred if the costs of probability sampling would be prohibitive and where feasibility issues become prohibitive.

Information on number of questionnaires returned was not collected because it is not a requirement of the methodology used here. The 2011 Young Men's Health Survey – and previous waves of research – adopted an in-home ‘random location’ with respondent self-completion and interviewer pick-up. Random location is the most common form for social and public policy research surveys as it combines a rigorous methodology with relatively low costs.

In this particular survey, we used Output Areas as the principal sampling unit. OAs are the base unit of the Census outputs and are based on groups of postcodes that fit within the boundaries of electoral wards/divisions and parishes. OAs represent the lowest geographical level on which full information can be generated through Census output. Each OA contains approximately 150 households and interviewers were required to achieve a target of 13 interviews (i.e. questionnaire pick-ups) in each OA.

A quota sheet was provided for each selected grouped OA, which reflects the actual composition of OA residents according to standard demographic criteria. Interviewers were required to interview a sample profile that exactly matches that of the grouped OA population profile using Census population information. This ensures that the sample is demographically representative at the micro-level, as well as geographically representative.

Crucially, interviewers were not required to record the number of questionnaires handed out, meaning it is not possible to record a response rate. This is in contrast to a random probability (pre-selected) survey where interviewers are required to visit randomly selected households and interview randomly selected individuals. With this technique, the response rate is recorded and taken as a measure of quality and robustness of the sample.

However, it is possible to work out an indicative response rate to the 2011 Young Men's Health Survey. ICM interviewers are required to leave a minimum of three addresses between calls, as well as include evening and weekend visits so that they maximise the chance of meeting their quotas and to avoid clustering in a single street. As such, it is reasonable to assume that interviewers distributed questionnaires to no more than a quarter of the households in each OA. This being the case, an approximate response rate would be around 35% (i.e. 13 completed surveys per point from 37.5 households visited). Nonetheless, this is an approximation and cannot be regarded as a true response rate.

RIM (Random Iterative Method) weighting is a technique commonly used to weight market research data to known targets, eg age groups regions, gender, and specifically to each variable (question) independently. The technique allows the analyst to adjust multiple characteristics in the dataset all at the same time in a way that ultimately keeps the different characteristics proportionate as a whole.

RIM weighting is a special form of target weighting. It can be a practical tool to use when there are targets (or populations) to which we wish the data for two or more variables, but not targets for the interlocking cells for these two or more variables. These are known as ‘rim weighting targets’. There may be more than two variables, which is where rim weighting is likely to be the chosen method. RIM weighting works by what is known as an iterative target weighting process. In other words, the software will calculate targets for the first rim and, after applying this weighting factor, it is highly improbable that the precise target percentages for one variable (e.g. gender) would be achieved. As the programme performs the iterations, the data gets closer and closer to the targets.

ICM uses Quantum, one of the most widely used tabulation and data packages in the survey research industry.

Translation by interviewers was not allowed which meant that to complete questionnaires the participants had to be English speakers

References

1. Singer M, *Introduction to Syndemics: A Critical Systems Approach to Public and Community Health*. 2009, San Francisco: Wiley.

2. Singer M, A dose of drugs, a touch of violence, a case of AIDS: Conceptualizing the Sava Syndemic*.* *Free Inquiry in Creative Sociology* 1996;24:99-110.

3. Wallace R, A Synergism of Plagues - Planned Shrinkage, Contagious Housing Destruction, and Aids in the Bronx*.* *Environmental Research* 1988;47:1-33.

4. P. F, Pathologies of power: Health, human rights and the new war on the poor*.* *North American Dialogue* 2003;6:1-4.

5. Singer M, Bulled N, Ostrach B, and Mendenhall E, Syndemics and the biosocial conception of health*.* *Lancet* 2017;389:941-950.

6. Tsai AC and Burns BFO, Syndemics of psychosocial problems and HIV risk: A systematic review of empirical tests of the disease interaction concept*.* *Social Science & Medicine* 2015;139:26-35.

7. Tsai AC, Mendenhall E, Trostle JA, and Kawachi I, Co-occurring epidemics, syndemics, and population health*.* *Lancet* 2017;389:978-982.

8. Coid J, Gonzalez Rodriguez R, Kallis C, et al., Ethnic disparities in psychotic experiences explained by area-level syndemic effects*.* *Br J Psychiatry* 2020;217:555-561.

9. Coid J, Zhang Y, Bebbington P, et al., A syndemic of psychiatric morbidity, substance misuse, violence, and poor physical health among young Scottish men with reduced life expectancy*.* *SSM Popul Health* 2021;15:100858.

10. Coid JW, Kirkbride JB, Barker D, et al., Raised incidence rates of all psychoses among migrant groups: findings from the East London first episode psychosis study*.* *Arch Gen Psychiatry* 2008;65:1250-8.

11. Coid JW, Ullrich S, Keers R, et al., Gang membership, violence, and psychiatric morbidity*.* *Am J Psychiatry* 2013;170:985-93.

12. Kirkbride JB, Fearon P, Morgan C, et al., Heterogeneity in incidence rates of schizophrenia and other psychotic syndromes: findings from the 3-center AeSOP study*.* *Arch Gen Psychiatry* 2006;63:250-8.

13. Wile, JUVENILE DELINQUENCY AND URBAN AREAS. A Study of Rates of Delinquents in Relation to Differential Characteristics of Local Communities in American Cities*.* *American Journal of Orthopsychiatry* 1943;13:176-176.

14. Faris R, Dunham H, *Mental Disorders in Urban Areas: An Ecological Study of Schizophrenia and Other Psychoses*. 1939: University of Chicago Press.

15. Bennetts A SL, Newman-Taylor K, What can experimental studies tell us about paranoia and anxiety? a systematic review with implications for theory and clinical practice*.* *Psychosis* 2021;14:162-175.

16. Ellett L, Varese F, Owens J, et al., Experimental studies of paranoid thinking in clinical and nonclinical populations: a systematic review and meta-analysis*.* *Psychol Med* 2023;53:5933-5944.

17. Skryabin VY, Martinotti G, Franck J, and Zastrozhin MS, Acute Alcoholic Hallucinosis: A Review*.* *Psychopathology* 2023.

18. Little R and D'mello D, A Cannabinoid Hypothesis of Schizophrenia: Pathways to Psychosis*.* *Innov Clin Neurosci* 2022;19:38-43.

19. Mcgrath J, Welham J, Scott J, et al., Association between cannabis use and psychosis-related outcomes using sibling pair analysis in a cohort of young adults*.* *Arch Gen Psychiatry* 2010;67:440-7.

20. Moore THM, Zammit S, Lingford-Hughes A, et al., Cannabis use and risk of psychotic or affective mental health outcomes: a systematic review*.* *Lancet* 2007;370:319-328.

21. Dean K and Murray RM, Environmental risk factors for psychosis*.* *Dialogues Clin Neurosci* 2005;7:69-80.

22. Ham S, Kim TK, Chung S, and Im HI, Drug Abuse and Psychosis: New Insights into Drug- induced Psychosis*.* *Experimental Neurobiology* 2017;26:11-24.

23. Fiorentini A, Cantu F, Crisanti C, et al., Substance-Induced Psychoses: An Updated Literature Review*.* *Front Psychiatry* 2021;12:694863.

24. Khantzian EJ, The self-medication hypothesis of addictive disorders: focus on heroin and cocaine dependence*.* *Am J Psychiatry* 1985;142:1259-64.

25. Cameron D and Jones IG, An epidemiological and sociological analysis of the use of alcohol, tobacco and other drugs of solace*.* *Community Med* 1985;7:18-29.

26. Karle A, Agardh A, Larsson M, and Arunda MO, Risky sexual behavior and self-rated mental health among young adults in Skane, Sweden - a cross-sectional study*.* *Bmc Public Health* 2023;23.

27. Gebeyehu DA and Mulatie M, Risky sexual behavior and its associated factors among patients with severe mental disorder in University of Gondar Comprehensive Specialized Hospital, 2018*.* *BMC Psychiatry* 2021;21:51.

28. Carey MP, Carey KB, Maisto SA, Gordon CM, and Vanable PA, Prevalence and correlates of sexual activity and HIV-related risk behavior among psychiatric outpatients*.* *Journal of Consulting and Clinical Psychology* 2001;69:846-850.

29. Park JW, Kim DJ, and Shin MH, The Effect of Stress on Compulsive Sexual Behavior Disorder: Active Coping Strategy and Self-Control as Mediators*.* *Psychiatry Investigation* 2021;18:997-1005.

30. Gh Y, *Psychology of Sex Addiction*. 2018, Seoul, Hakjisa.

31. Fazel S, Gulati G, Linsell L, Geddes JR, and Grann M, Schizophrenia and violence: systematic review and meta-analysis*.* *PLoS Med* 2009;6:e1000120.

32. Ltd. C, *The ACORN user guide: The Consumer Classification.* 2014, London: CACI Ltd.

33. Jeremy W Coid SU, Constantinos Kallis, Mark Freestone, Rafael Gonzalez, Laura Bui, Artemis Igoumenou, Anthony Constantinou, Norman Fenton, William Marsh, Min Yang, Bianca Destavola, Junmei Hu, Jenny Shaw, Mike Doyle, Laura Archer-Power, Mary Davoren, Beatrice Osumili, Paul Mccrone, Katherine Barrett, David Hindle, and Paul Bebbington., *Improving Risk Management for Violence in Mental Health Services: A Multi-Methods Approach*. 2016, Southampton (UK): NIHR Journals Library.
